# Design Principles for Engineering Ionic Liquid-Gold Nanoparticles for Therapeutic Delivery to the Brain

**DOI:** 10.1021/acsnano.5c02375

**Published:** 2025-07-03

**Authors:** Talia A. Shmool, Laura K. Martin, Andreas Jirkas, Sophie V. Morse, Claudia Contini, Yuval Elani, Jason P. Hallett

**Affiliations:** a Department of Chemical Engineering, 4615 Imperial College London, South Kensington Campus, London SW7 2AZ, U.K.; b Department of Engineering Science, University of Oxford, Parks Road, Oxford OX1 3PJ, U.K.; c Department of Bioengineering, 4615 Imperial College London, South Kensington Campus, London SW7 2AZ, U.K.; d U.K. Dementia Research Institute at Imperial College London, White City Campus, London W12 0BZ, U.K.; e Department of Life Sciences, 4615 Imperial College London, South Kensington Campus, London SW7 2AZ, U.K.

**Keywords:** therapeutic delivery, gold nanoparticles, ionic liquids, amino acids, focused ultrasound, blood−brain barrier

## Abstract

Ionic liquid (IL) nanotechnology holds significant promise for designing nanoscale materials with tunable viscosity, polarity, and thermal stability for advanced therapeutic applications. However, the field currently lacks comprehensive guidelines for integrating ILs into complex therapeutic formulations. Herein, we propose the key design considerations for engineering immunoglobulin G (IgG) conjugated to gold nanoparticles (AuNPs) in the presence of choline-based ILs. By judicious IL cation and anion selection, we fine-tune the supramolecular assemblies and leverage the unique physicochemical properties of ILs to impart AuNPs with advantageous characteristics including enhanced structural, thermal, and thermodynamic stabilities, highly tunable morphologies, and markedly reduced aggregation propensities. Through systematic circular dichroism measurements, the thermodynamic parameters of the complex formulations were determined, offering insight into the IgG conformational changes and design parameters for systems of enhanced IgG conjugation to AuNP surfaces. In demonstrating the power of our design approach, the complex formulation of IgG-choline chloride-AuNPs, also including trehalose, histidine, and arginine, was delivered via focused ultrasound and microbubbles across the blood–brain barrier and showed a 7.6-fold increase in delivery *in vivo* compared to the traditional formulation. We demonstrate that IgG-IL-AuNPs can be easily and precisely manipulated at the nanometer scale, enabling the formation of versatile structural configurations. Holistically, we believe the rational design approach developed will advance the engineering of tailored IL-nanocarriers for targeted therapeutic delivery and broaden the scope of IL applications in biomedicine.

## Graphical abstract

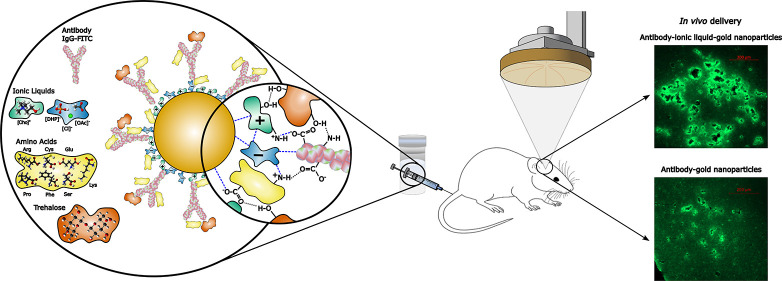

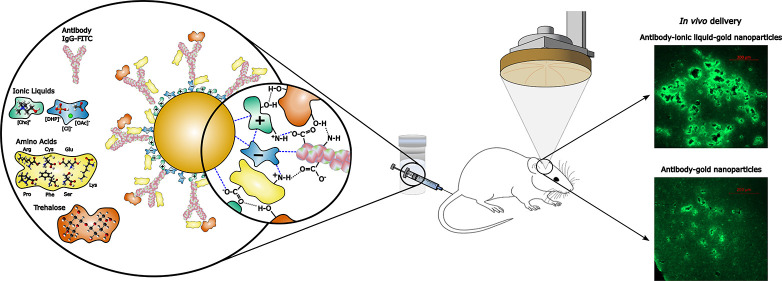

## Introduction

Ionic liquid (IL) nanotechnology is a nascent field, including the design and engineering of IL-nanoscale materials via a self-assembly approach. ILs are compounds composed of cations and anions, offering unique features including tunable viscosity, polarity, and hydrophobicity and enhanced thermal, structural, and thermodynamic stabilities.^1–3^ As such, judicious selection of biocompatible IL cations and anions^4,5^ can be exploited in molecular design for fine-tuning the self-assembly of novel functional nanomaterials and biomaterials.^6^

While the field of IL nanotechnology is in its infancy, several studies have examined IL structures and functions for therapeutic applications.^7–10^ Biocompatible ILs have been explored as building blocks for engineering-tailored nanoarchitectures for therapeutic delivery applications.^11–13^ Previous research has demonstrated that diverse imidazolium-based ILs can enhance drug solubility and transdermal delivery and promote the surface functionalization of metal nanoparticles (NPs) for therapeutic applications.^14–17^ However, imidazolium-based ILs are of high viscosity, poor biocompatibility, and low biodegradability and can promote protein aggregation,^17^ limiting the exploitation of these ILs in pharmaceutical applications. Biocompatible choline-based ILs have been shown to improve therapeutic thermostability, drug solubility, bioavailability, and topical and transdermal drug delivery.^10,18–21^ NPs for targeted therapeutic delivery typically require surface functionalization with diverse targeting agents and moieties^22^ and chemical modifications of the installed motif and the NPs. This increases the synthetic complexity and limits amendable NP classes and applications. In IL-NP designs, the NPs can consist of lipids, polymers, and inorganic materials, and the ILs can serve as the major frame of the structure to facilitate intermolecular conjugation of peptide and protein molecules to the IL-NP surfaces. This offers opportunities for developing multifunctional NPs for therapeutic delivery via electrostatic conjugation of the therapeutic to the surfaces of NPs, as directed by the IL ions. However, across the IL nanotechnology field, the key design considerations for using ILs as building blocks in complex therapeutic formulations have yet to be comprehensively outlined and require further study.

Herein, we develop a rational thermodynamic-guided approach and outline the key design considerations for utilizing ILs as building blocks for the self-assembly of immunoglobulin G (IgG)-IL-gold nanoparticles (AuNPs) entrapped in a matrix of disaccharide and amino acid molecules. We judiciously selected the biocompatible ILs choline acetate ([Cho]­[OAc]), choline dihydrogen phosphate ([Cho]­[DHP]), and choline chloride ([Cho]­[Cl]) for the construction of a library of IL-AuNPs. On the basis of the physicochemical and structural properties, these biocompatible ILs can facilitate electrostatic conjugation of the IgG molecules to the IL-AuNP surfaces.^23,24^ These ILs have also been shown to improve the structural and thermodynamic stabilities of therapeutic formulations, which could aid in enhancing *in vivo* delivery.^10,18,25,26^ We utilize AuNPs as the model inorganic NP platform. AuNPs have shown great promise for site-specific therapeutic delivery applications, particularly across the blood–brain barrier (BBB),^27^ our selected target site, which poses significant delivery challenges. Notably, focused ultrasound (FUS) in combination with microbubbles is a powerful technique for brain-targeted therapeutic delivery.^28–30^ Ultimately, we determine the key complex formulation elements for achieving enhanced thermodynamic, thermal, and structural stabilities of IgG-IL-AuNPs and improved FUS-mediated delivery to the brain.^31,32^

In each complex formulation, trehalose, histidine,^33–39^ and a select amino acid, specifically arginine (Arg),^40,41^ cysteine (Cys),^40,42^ glutamic acid,^40,43^ lysine (Lys),^40,44–47^ phenylalanine (Phe),^40,44,45^ proline (Pro),^40,47^ and serine (Ser)^40,48^ (Tables S1 and S2), are included at proportions previously associated with suppressed therapeutic aggregation for enhanced delivery across the BBB.^34,38–40,42,44,45^ The model therapeutic IgG-fluorescein isothiocyanate (FITC) is incorporated with the IL-AuNPs at proportions found to provide surface coverage and increase the structural and thermodynamic stabilities of the system.^18,49–51^ Notably, complex formulations composed of these ILs, AuNPs, disaccharides, amino acids, and IgG molecules have yet to be designed.

We conduct dynamic light scattering (DLS), zeta potential, and temperature variable circular dichroism (CD) spectroscopy measurements to determine the aggregation propensity, surface charge, and the structural, thermal, and thermodynamic stabilities of the complex formulations. We perform transmission electron microscopy (TEM) to examine the conjugation of the IgG molecules to the surfaces of the IL-AuNPs. Finally, we deliver the lead complex formulation presenting reduced aggregation propensity and increased structural, thermal, and thermodynamic stabilities across the BBB via FUS and microbubbles. Overall, we set out to present a thermodynamic-guided approach and outline the key design considerations to engineer IL-AuNPs. Additionally, we aim to highlight the role of IL intermolecular interactions in IgG-IL-AuNPs, to ultimately predict the self-assembly processes and advance the engineering of IL-NP structures offering suppressed aggregation, electrostatic conjugation, structural, thermal, and thermodynamic stabilities, and enhanced delivery and accumulation in the brain *in vivo.*

## Results and Discussion

### Design and Engineering Complex Formulations

On the basis of thermodynamic principles, IL-AuNP construction would involve the ILs facilitating hydrogen bonding and electrostatic interactions and promoting the conjugation of IgG molecules onto the IL-AuNP surfaces. This would result in the self-assembly of IgG-IL-AuNPs of tunable physicochemical, morphological, and thermodynamic properties (Figure 1A,B).^24^ As such, the integrated disaccharide, histidine, and the select amino acid molecules would serve as a matrix surrounding the IgG-AuNPs and IgG-IL-AuNPs and introduce additional multiple intermolecular interactions for suppressed aggregation propensity. Understanding the synergistic hydrogen bonding and electrostatic interactions and the influence of the diverse amino acids on the aggregation propensity and binding of the IgG molecules onto IL-AuNP surfaces^23,52^ is key in the development of a robust thermodynamic-guided methodology for designing complex formulations of IgG-IL-AuNPs (Figure 1C). Notably, exploring the impact of the tunable attributes of the IgG-IL-AuNPs on *in vivo* delivery efficacy is critical, as this informs the engineering and optimization of complex formulations in an increasingly specified manner for therapeutic delivery applications (Figure 1D).

**Figure 1:**
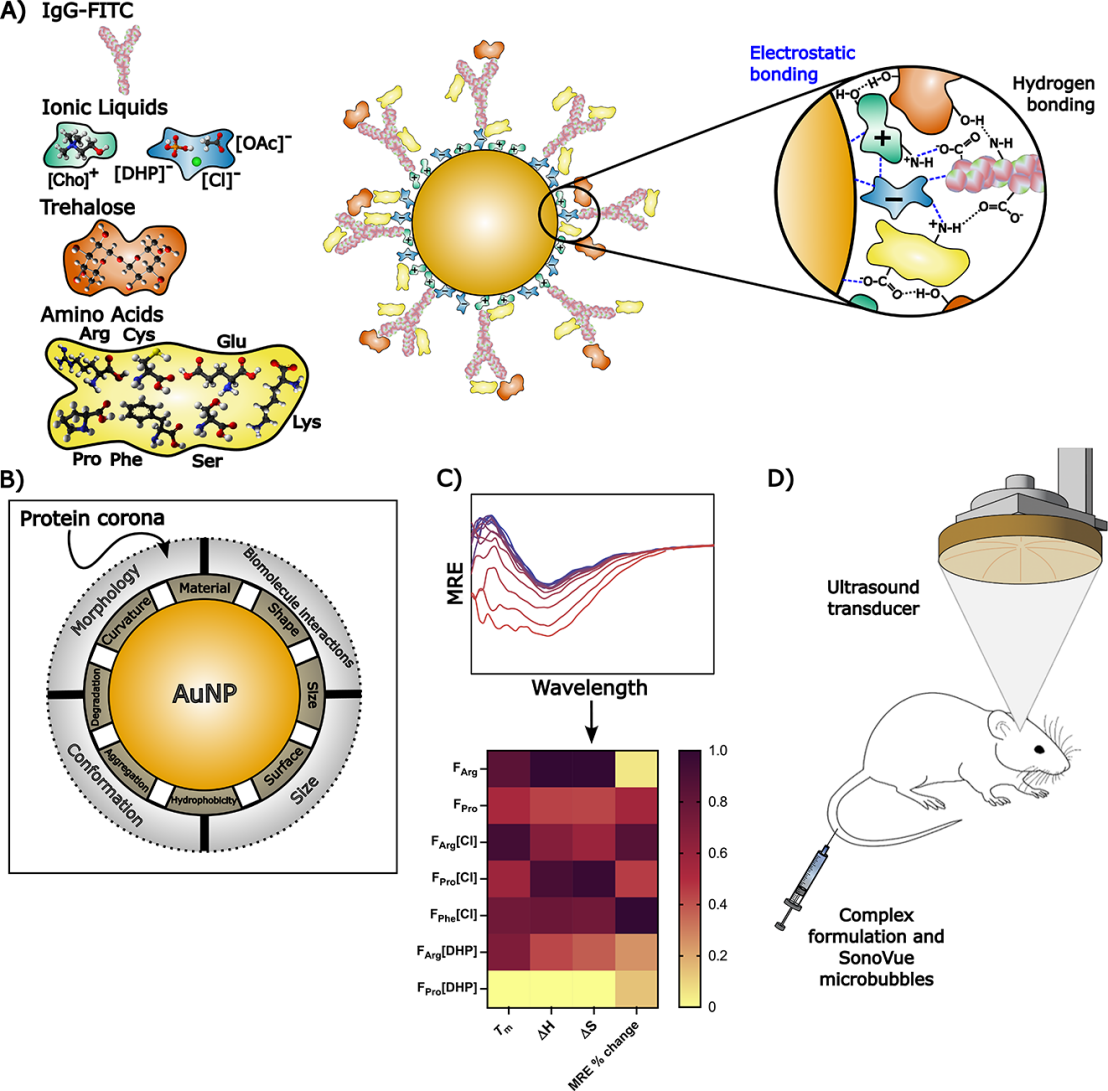
Illustration of the key components of this research. (A) Design and engineering of IgG-IL-AuNPs, with ILs promoting hydrogen bonding and electrostatic conjugation of IgG molecules onto the IL-AuNP surfaces. The disaccharide and amino acid molecules synergistically induce confinement effects for improved IgG-IL-AuNP structural, thermal, and thermodynamic stabilities. (B) Attributes of IL-AuNPs, which can be modified during complex formulation design to yield the formation of protein coronas on the IL-AuNP surfaces, as characterized via TEM experiments. (C) Representative mean residual ellipticity (MRE) spectrum of an IgG-IL-AuNP complex formulation from which the thermodynamic parameters of the system can be calculated and applied in design optimization. (D) *I* *n vivo* delivery of IgG-IL-AuNPs across the blood–brain barrier utilizing focused ultrasound and microbubbles.

### Examining Aggregation Suppression and Complex Formulation Surface Charge

The IgG-AuNPs in the complex formulations were examined by DLS measurements and consistently showed relatively higher hydrodynamic diameter (*D*_h_) and polydispersity index (PDI) values compared to complex formulations containing IgG-IL-AuNPs (Figure 2 and Tables S1 and S2). Additionally, contingent on the amino acid, we found that the *D*_h_ values for the IgG-AuNPs varied. In the absence of IL, the *D*_h_ values increased in the order arginine (F_Arg_), proline (F_Pro_), serine (F_Ser_), phosphate buffered saline (F_PBS_), lysine (F_Lys_), cysteine (F_Cys_), glutamic acid (F_Glu_), and phenylalanine (F_Phe_) (Table S3).

**Figure 2:**
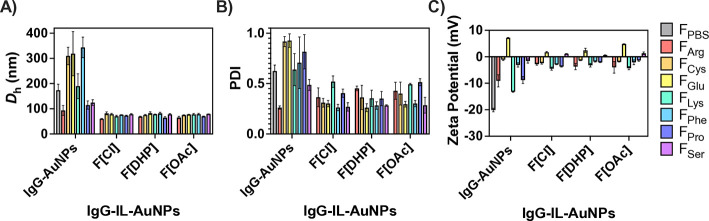
(A) Hydrodynamic diameter (*D*_h_), (B) polydispersity index (PDI), and (C) zeta potential values of complex formulations containing IgG-AuNPs and IgG-IL-AuNPs (Tables S2 and S3). Each complex formulation included trehalose, histidine, and a select amino acid. The ILs included are [Cho]­[Cl] (F­[Cl]), [Cho]­[DHP] (F­[DHP]), and [Cho]­[OAc] (F­[OAc]). Also shown is IgG-AuNPs in phosphate buffered saline (F_PBS_).

Overall, the IgG-[Cho]­[Cl]-AuNPs showed lower *D*_h_ values, increasing for IgG-[Cho]­[OAc]-AuNPs, and highest for IgG-[Cho]­[DHP]-AuNPs. Distinctly, this trend was reversed for the IgG-IL-AuNPs in F_Cys_ and F_Pro_. Notably, IgG-IL-AuNPs in F_Arg_ displayed the lowest *D*_h_ values, indicating the greatest aggregation suppression. Additionally, the *D*_h_ values were raised for the IgG-IL-AuNPs in the increasing order of F_Lys_, F_Pro_, F_Glu_, F_Cys_, F_Ser_, and F_Phe_. Furthermore, we found consistently lower PDI values for IgG-IL-AuNPs in F_Phe_, F_Ser_, and F_Glu_, increasing for F_Cys_ and F_Arg_, and highest for F_Pro_ and F_Lys_. This data evidences the power of ILs as building blocks for constructing complex formulations of IL-AuNPs of suppressed aggregation propensity and controlled *D*_h_ and PDI values, which are favorable for intravenous delivery applications.

We found that the IgG-AuNPs in F_PBS_ presented the most negative zeta potential value of the systems examined. Overall, IgG-IL-AuNPs and IgG-[Cho]­[Cl]-AuNPs exhibited more negative zeta potential values, while IgG-[Cho]­[OAc]-AuNPs presented more positive zeta potential values. Additionally, the zeta potential values were more negative for the IgG-AuNPs and the IgG-IL-AuNPs in F_Lys,_ F_Pro_, and F_Arg_, less negative for F_Cys_ and F_Phe_, and positive zeta potential values were observed for F_Ser_ and F_Glu_. Notably, for a complex formulation of a given amino acid, the zeta potential value of the IgG-IL-AuNPs was of lower magnitude compared to that of the IgG-AuNPs.

### Studying IgG Secondary Structure and Conformational Changes

Systematically examining the mean residual ellipticity (MRE) spectrum of each system (Figure 3 and Figure S1) provided knowledge regarding the key structural features, predictive self-assembly, IgG folding attributes, and conformational changes in the complex formulations. MRE spectra for the IgG-AuNPs in F_PBS_, F_Arg_, F_Pro_, and IgG-IL-AuNPs in F_Arg_[DHP], F_Pro_[DHP], F_Arg_[Cl], and F_Pro_[Cl] each displayed a β-sheet-rich structure for the IgG molecules, indicated by the distinct negative absorbance peak at 218 nm^53^ and positive peak at approximately 200 nm. The near-native IgG structure and suppressed aggregation of the IgG-IL-AuNPs determined via DLS measurements suggest greater structural and colloidal stabilities of the IgG-IL-AuNPs.^54–56^ However, in each case, these features gradually degraded upon heating from 25 to 95 °C, attributed to thermal denaturation and the resulting relatively lower structural and colloidal stabilities of these IgG-IL-AuNPs.

**Figure 3:**
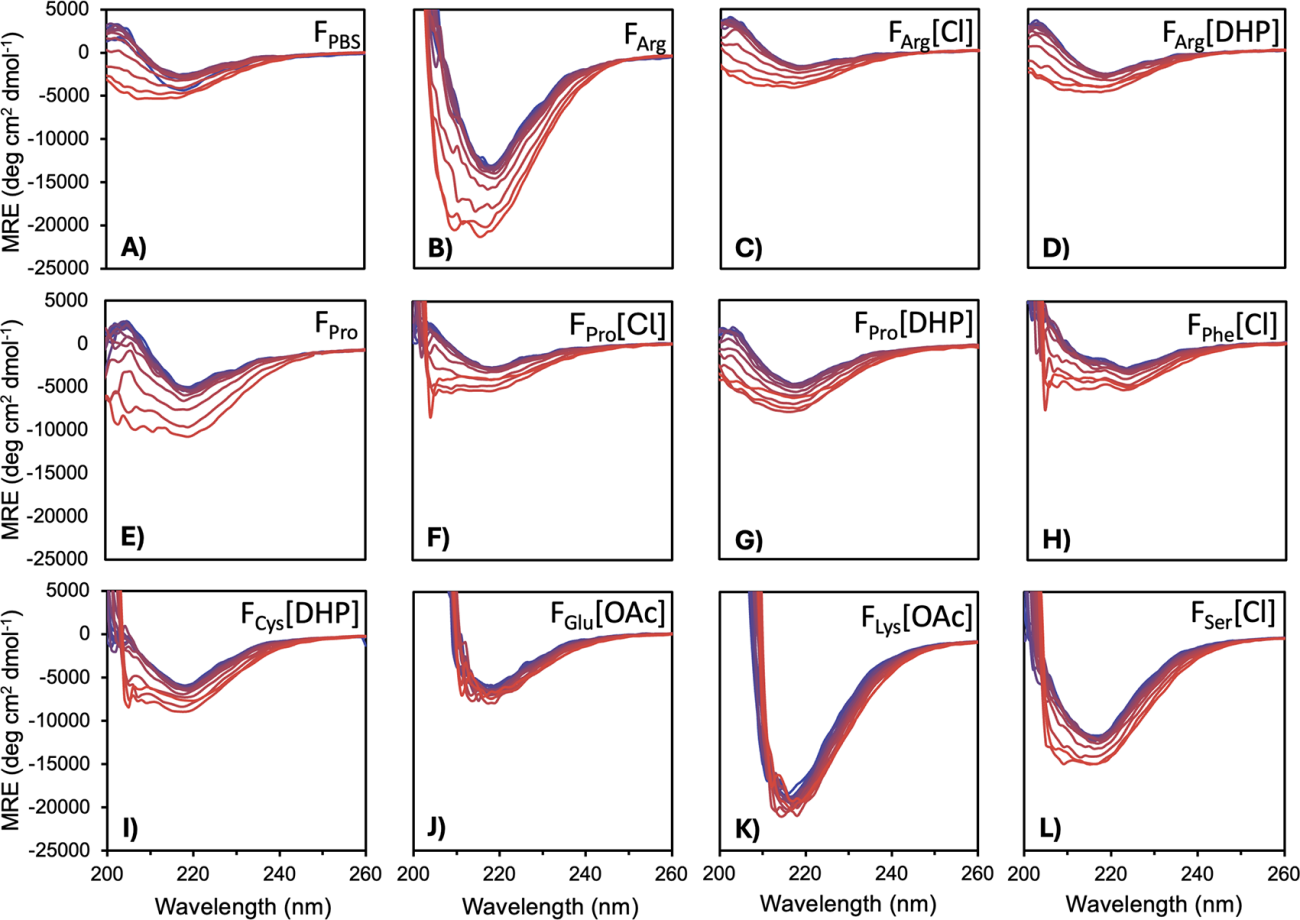
MRE spectra derived from the temperature variable CD data for IgG-AuNPs in F_PBS_ and IgG-AuNPs and IgG-IL-AuNPs in complex formulations. Spectra were measured from 200 to 260 nm with the temperature increasing from 25 °C (blue) to 95 °C (red) in 5 °C increments. See Figure S1 for MRE spectra of additional systems developed. IgG-AuNPs in (A) F_PBS_ and (E) F_Pro_ and IgG-IL-AuNPs in (C) F_Arg_[Cl], (D) F_Arg_[DHP], (F) F_Pro_[Cl], and (G) F_Pro_[DHP] show a distinct negative absorbance peak at 218 nm indicating β-sheet-rich IgG structures. This feature is reduced in (B) F_Arg_ and (H) F_Phe_[Cl] and absent in (I) F_Cys_[DHP], (J) F_Glu_[OAc], (K) F_Lys_[OAc], and (L) F_Ser_[Cl], indicating aggregate assemblies.

The MRE spectra of IgG-AuNPs in F_Cys_ and F_Phe_ and IgG-IL-AuNPs in F_Cys_[Cl] and F_Cys_[DHP] suggested that aggregation of the β-sheet structure was present prior to heating (Figure 3 and Figure S1). For the IgG-IL-AuNPs in F_Phe_[Cl] and F_Phe_[DHP], we observed a double negative peak at 25 °C between 205 and 225 nm, indicative of α-helices in the secondary structure. In these cases, we also observed a significant loss of spectral features upon heating, evidencing a degree of secondary structural changes due to thermal denaturation. Conversely, IgG-AuNPs in F_Cys_ and F_Phe_ as well as IgG-AuNPs and IgG-IL-AuNPs in F_Lys_, F_Glu_, and F_Ser_ exhibited significantly deeper negative peaks at 218 nm and relatively minimal changes with heating (Figure 3 and Figure S1). Based on previous work,^10,18^ it is expected that these complex formulations include β-sheet-rich aggregate structures at 25 °C and present thermal denaturation resistance. The IgG-[Cho]­[OAc]-AuNPs showed limited β-sheet content and negligible structural changes upon heating, also indicating the presence of thermostable aggregate assemblies.

### Evaluating the Protein Corona on IL-AuNP Surfaces

We performed TEM experiments to further examine the nanoassemblies of IgG-AuNPs and IgG-IL-AuNPs, which exhibited relatively low *D*_h_ and PDI values and varying degrees of the β-sheet structure (Figure 4). For the IgG-IL-AuNPs, we found protein coronas on the surfaces of the [Cho]­[Cl]-AuNPs in F_Arg_, F_Lys_, F_Pro_, and F_Ser_ and the [Cho]­[OAc]-AuNPs in F_Lys_. In contrast, we failed to observe protein coronas on the surfaces of the [Cho]­[DHP]-AuNPs and the IgG-AuNPs and IgG-IL-AuNPs in F_Glu_ and F_Phe_. Additionally, the IgG-AuNPs in F_Arg_ presented an irregular-shaped protein corona,^57^ and the IgG-AuNPs in F_PBS_ lacked a measurable protein corona on the AuNP surfaces. This was attributed to colloidal aggregation on the TEM grids.^58,59^ Additionally, the TEM and CD experiments showed that IgG-IL-AuNPs in F_Lys_[OAc] and F_Lys_[Cl] presented the highest aggregation propensity. Likely, the IgG-IL-AuNPs were entrapped to varying degrees in the trehalose and amino acid matrices, and this influenced the spontaneous self-assembly, intermolecular interactions, and electrostatic conjugation of the IgG-IL-AuNPs, which ought to be accounted for in IL-AuNP design.

**Figure 4:**
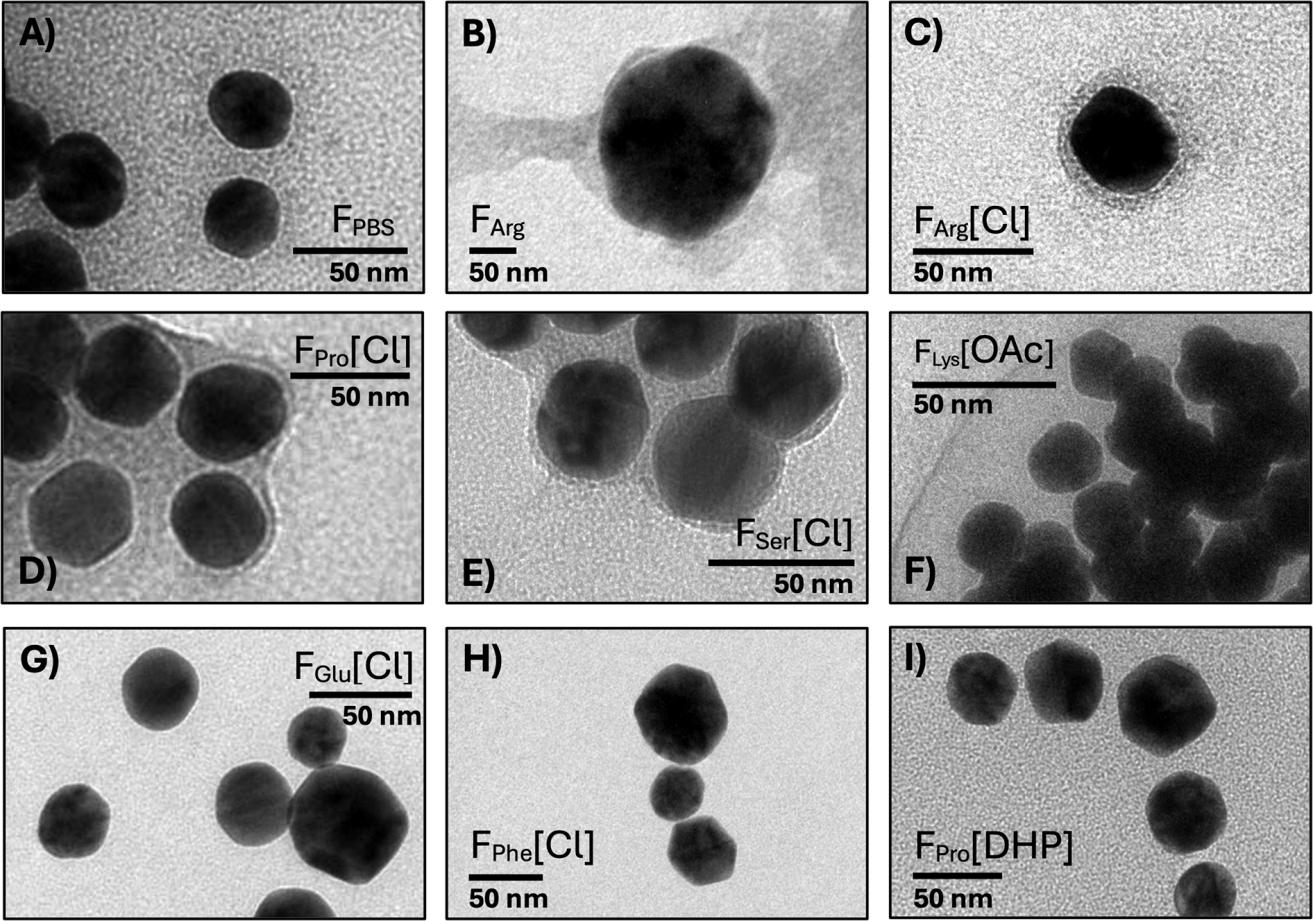
TEM micrographs of IgG-AuNPs in (A) F_PBS_ and (B) F_Arg_, and complex formulations of IgG-IL-AuNPs. See Figure S2 for micrographs of additional complex formulations. Shown are protein coronas on the surfaces of the [Cho]­[Cl]-AuNPs in (C) F_Arg_, (D) F_Pro_, and (E) F_Ser_ and [Cho]­[OAc]-AuNPs in (F) F_Lys_. The protein corona is absent on the surfaces of the [Cho]­[Cl]-AuNPs in (G) F_Glu_ and (H) F_Phe_ and on the surfaces of (I) [Cho]­[DHP]-AuNPs in F_Pro_.

### Thermodynamic Properties of Complex Formulations

We next determined the relative thermodynamic properties of the IgG-AuNPs and IgG-IL-AuNPs, providing insight into the self-assembly and structural, thermal, and colloidal stabilities of the systems. Specifically, we derived and calculated the melting temperature (*T*_m_) and change in enthalpy (Δ*H*) and entropy (Δ*S*) from the temperature variable CD data of the IgG-IL-AuNPs and IgG-AuNPs, as previously described.^10,26^ F_Arg_[Cl], F_Arg_[DHP], F_Pro_[Cl], and F_Pro_[DHP] were chosen for analysis as these exhibited relatively high structural and colloidal stabilities and low *D*_h_ and PDI values. For contrast, F_Cys_[Cl], F_Cys_[DHP], F_Phe_[Cl], F_Phe_[DHP], and the IgG-AuNPs in F_PBS_, F_Arg_, and F_Pro_ were also evaluated, as these showed a degree of β-sheet secondary structure, yet displayed distinct structural features and relatively higher *D*_h_ values indicative of greater aggregation propensity.

Overall, the *T*_m_ values of the complex formulations examined were approximately 80 °C (Table S4), reflecting the thermostable nature of the developed systems. The lowest *T*_m_, Δ*H*, and Δ*S* values were found for the IgG-IL-AuNPs in F_Phe_[DHP] and F_Pro_[DHP] (5). This can be linked to the lack of protein coronas on the surfaces of the [Cho]­[DHP]-AuNPs. We consider that the thermodynamic stability is predictive of IgG conjugation to the IL-AuNP surfaces. Notably, the IgG-IL-AuNPs in F_Arg_[DHP] also possessed relatively low Δ*H* and Δ*S* values (Figure 5). Conversely, the IgG-IL-AuNPs in F_Arg_[Cl] and F_Pro_[Cl] both showed protein coronas on the surfaces of the [Cho]­[Cl]-AuNPs and exhibited significantly higher *T*_m_, Δ*H*, and Δ*S* values, as well as F_Phe_[Cl]. These trends were mirrored by the relative change in the MRE spectrum of each system due to heating. Based on the MRE spectra, the IgG-[Cho]­[Cl]-AuNPs displayed a greater degree of conformational changes compared to equivalent IgG-[Cho]­[DHP]-AuNP systems. Interestingly, the IgG-IL-AuNPs in F_Cys_[Cl] and F_Cys_[DHP] displayed minimal changes in MRE spectral features with heating and demonstrated relatively high *T*_m_, Δ*H*, and Δ*S* values. In contrast, the thermodynamic parameters and conformational changes of the IgG-AuNPs in F_PBS_, F_Arg_, and F_Pro_ were similar to those of the IgG-[Cho]­[Cl]-AuNPs. Nonetheless, these systems displayed significantly greater aggregation propensities, as exemplified by the relatively high *D*_h_ and PDI values.

**Figure 5:**
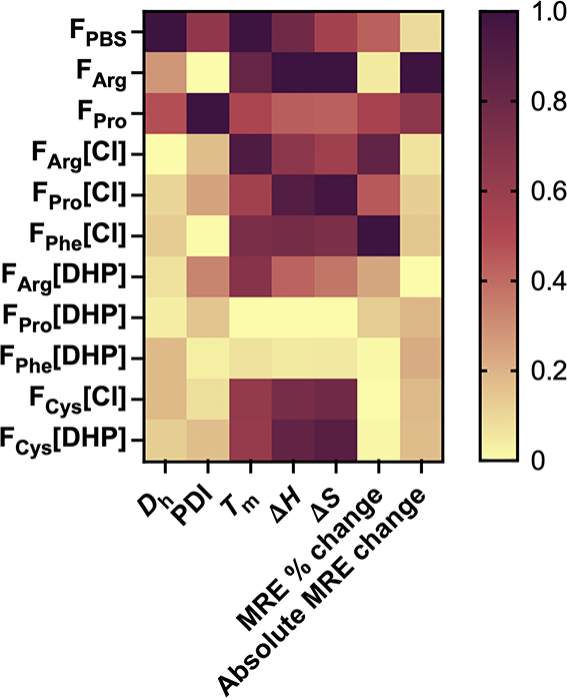
Heatmap showing the hydrodynamic diameter (*D*_h_) and polydispersity index (PDI) values found via DLS measurements, and the melting temperature (*T*_m_), change in enthalpy (Δ*H*) and entropy (Δ*S*), relative change in MRE, and absolute change in MRE for the systems exhibiting native and partial secondary structures, as determined via CD spectroscopy measurements. To aid comparison and reveal correlations between parameters, values were scaled from 0 to 1 relative to the range of each variable (absolute values in Table S4).

### *In Vivo* Targeted Delivery of Complex Formulations across the BBB

On the basis of the reduced *D*_h_ and PDI values, visible protein corona, and enhanced structural, thermal, and thermodynamic stabilities, the complex formulation of IgG-[Cho]­[Cl]-AuNPs in F_Arg_ was selected for FUS-mediated delivery *in vivo* (Figure 6A). Evidence of successful enhanced permeability of the BBB in the left hippocampus (Figure 6B) was observed via the detection of signal from the fluorescently labeled compounds (Figure 6C–E). The opposite right hippocampus, lacking ultrasound, served as a control region, for which fluorescence signal was not detected in the mouse brains. The normalized optical density (NOD) quantification calculated from the obtained images showed that significantly higher *in vivo* delivery was observed for IgG-[Cho]­[Cl]-AuNPs in F_Arg_ (21.6 ± 11.2) compared to the IgG-AuNPs in F_Arg_ lacking [Cho]­[Cl] (8.01 ± 4.23) and IgG-FITC in PBS (2.82 ± 1.34). The distribution of the delivered compounds, detected immediately following sonication, was found to be concentrated around the blood vessels in a spot-like pattern, as opposed to uniformly distributed throughout the parenchyma.

**Figure 6:**
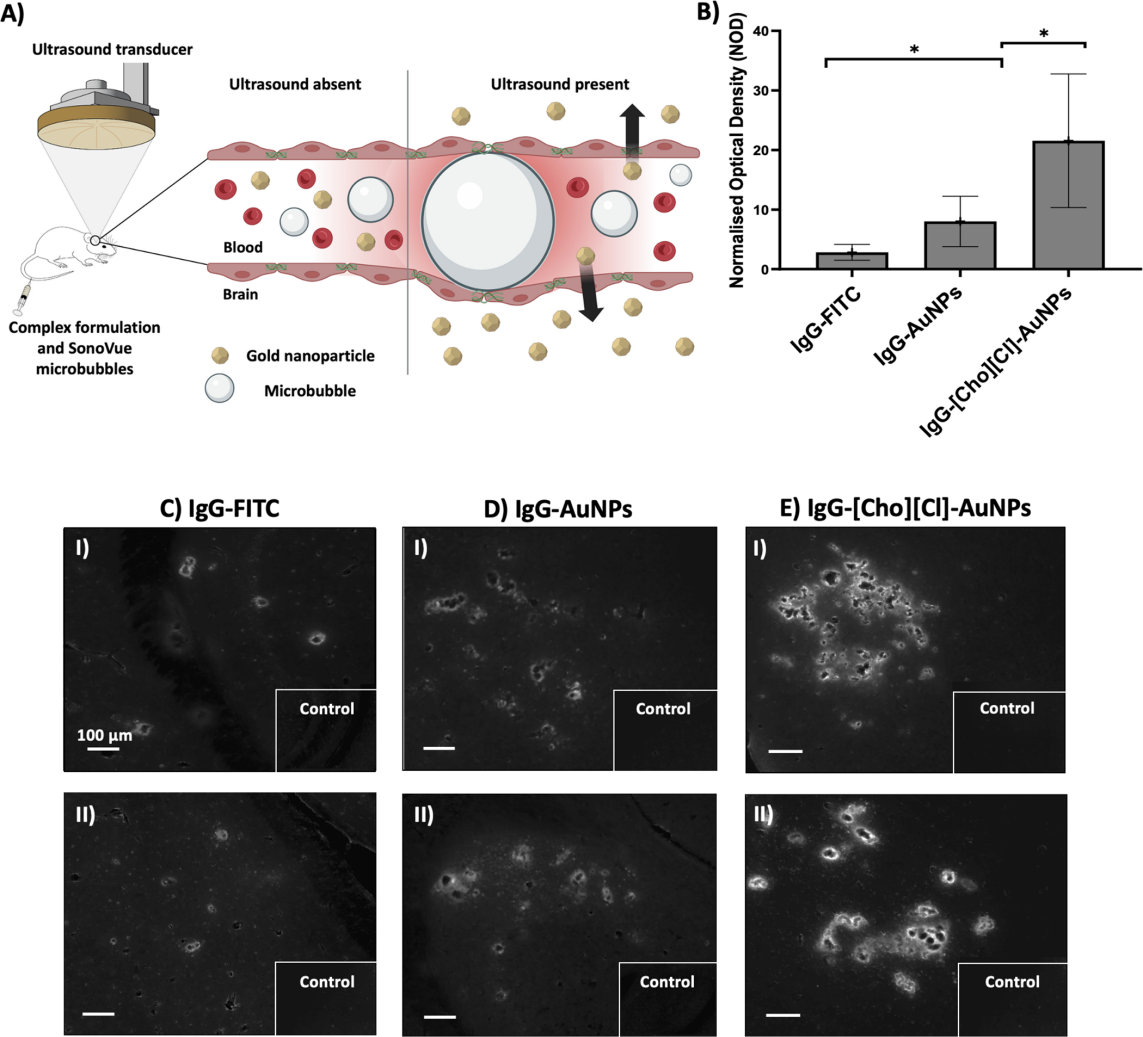
(A) Schematic showing the *in vivo* FUS-mediated delivery of AuNPs into the brain. (B) Fluorescence from the compounds delivered to the brain was quantified by NOD. Fluorescence images (C–E) show the detection of signal from the fluorescently labeled compounds in the left targeted hippocampi and the right hippocampi (control) upon delivering IgG-FITC in PBS, IgG-AuNPs in F_Arg_ lacking IL, and IgG-[Cho]­[Cl]-AuNPs in F_Arg_. I and II show two biological replicates for each condition.

### Understanding the Design and Thermodynamic Principles to Engineer Complex Formulations of IgG-IL-AuNPs

Judicious IL and amino acid selection implemented during the design process allow for engineering controlled nanoassemblies of IgG-IL-AuNPs. Based on the experimental and thermodynamic data obtained, we propose that the presence of ILs promoted the conjugation of IgG molecules onto the AuNP surfaces by mediating an extended network of electrostatic and hydrogen bonding interactions. Conversely, the relatively high *D*_h_ and PDI values and lack of protein coronas in the IgG-AuNP systems lacking ILs further highlight the role of the IL ions in facilitating intermolecular interactions contributing to enhanced structural, thermal, and thermodynamic stabilities and IgG conjugation to AuNP surfaces. Notably, this is with the exception of the IgG-AuNPs in F_Arg_, which exhibited relatively low *D*_h_ and PDI values, yet an irregular-shaped protein corona. This is attributed to the positive charge surrounding the planar guanidinium groups of the arginine molecules, contributing to electrostatic interactions, a dominant driving force in peptide association,^60^ and amplified upon IL inclusion.

We postulate that [Cho]­[Cl]-AuNPs mediate the electrostatic interactions in F_Arg_ and F_Lys_, leading to the conjugation of IgG molecules to the surfaces of [Cho]­[Cl]-AuNPs. In contrast to the arginine and lysine molecules, the relatively more compact structure of the serine molecules could result in reduced crowding and confinement effects. Consequently, IgG-AuNPs in F_Ser_ possess a greater aggregation propensity and limited thermodynamic and structural stabilities. Nonetheless, the protein corona observed on the surfaces of the [Cho]­[Cl]-AuNPs in F_Ser_ reflects that [Cho]­[Cl] effectively mediates electrostatic conjugation of the IgG molecules to the AuNP surfaces. Likely, the proline molecules also facilitate strong hydrophobic interactions with the aggregation prone and surface exposed hydrophobic residues of the IgG molecules. This could result in suppressed aggregation propensity and improved structural and thermodynamic stability of the IgG-AuNPs in F_Pro_.^61^ Alike, we observed the postulated electrostatic conjugation of the IgG molecules to the surfaces of the [Cho]­[Cl]-AuNPs in F_Pro_. Conversely, F_Glu_ showed limited structural and thermodynamic stability, and the relatively more positive surface charge on the AuNPs and IL-AuNPs, in the presence of glutamic acid molecules, would explain the absent protein corona.^62^ This could also be attributed to steric repulsions of the glutamic acid chains and disruption of the intermolecular hydrogen bond network in F_Glu_. Similarly, steric hindrance and hydrophobic effects could result in the lack of electrostatic conjugation of IgG molecules to the surfaces of the IL-AuNPs in F_Phe_.^63–66^

Compared with the IgG-[Cho]­[Cl]-AuNPs, we failed to observe conjugation of the IgG molecules onto the surfaces of the [Cho]­[DHP]-AuNPs, regardless of the amino acid integrated. This could be attributed to the previously identified steric effects and disruption of the intermolecular interactions of [Cho]­[DHP] inclusive therapeutic formulations.^10,19^ Notably, the steric effects of the IL-AuNPs are a key design consideration for electrostatic conjugation of IgG molecules to IL-AuNP surfaces.

Overall, we found limited conjugation of the IgG molecules to the surfaces of [Cho]­[OAc]-AuNPs; however, F_Lys_[OAc] and F_Cys_[OAc] displayed protein coronas on the surfaces of the IL-AuNPs. We consider that the tetrahedral ammonium cations and thiol groups of the lysine and cysteine molecules, respectively, could act as hydrogen bond donors to the acetate anions, thereby facilitating a symmetric charge distribution and strong network of electrostatic and hydrogen bonding interactions.^67–72^ This could promote the electrostatic conjugation of the IgG molecules to the surfaces of the IgG-IL-AuNPs in F_Lys_ and F_Cys_.^73–75^ Additionally, the DLS and CD spectroscopy measurements demonstrated significant aggregation for the IgG-IL-AuNPs in F_Arg_[OAc], which was suppressed for the IgG-IL-AuNPs in F_Arg_[Cl]. Likely, for the IgG-IL-AuNPs in F_Arg_[OAc], delocalization of the positive charge surrounding the planar guanidinium groups of the arginine molecules results in disruption of the intricate intermolecular interactions of the IgG-[Cho]­[OAc]-AuNPs. This could lead to conformational changes and thereby a greater aggregation propensity of the IgG-IL-AuNPs in F_Arg_[OAc]. Similarly, the IgG-IL-AuNPs in F_Pro_[OAc] lacked a protein corona despite the IgG-IL-AuNPs in F_Pro_[Cl] demonstrating conserved secondary IgG structure and protein corona formation. We consider that the solely available proton for hydrogen bonding in the proline molecule is that of the amide group, and the conformational rigidity of the proline side chain could restrict hydrogen bonding with the acetate anions due to the fixed bonding angles and steric bulk. This further emphasized that the electrostatic conjugation of the IgG molecules onto the IL-AuNP surfaces in F_Pro_[Cl], as opposed to F_Pro_[OAc], is largely directed via the IL anions, and enhanced structural, thermal and thermodynamic stability, and self-assembly is induced contingent on the identity of the IL ions and amino acid molecules.

We suggest that the restricted aggregation and greater structural, thermal, and thermodynamic stability of the IgG-IL-AuNPs in F_Arg_[Cl] resulted in the improved FUS-mediated delivery across the BBB compared to the IgG-AuNPs. The *in vivo* delivery achieved for the IgG-IL-AuNPs is in agreement with previous work showing FUS-mediated delivery of agents, above 60 nm, across the BBB.^76–80^ However, for the first time, we observed significantly enhanced FUS-mediated delivery of IgG-IL-AuNPs compared to IgG-AuNPs in F_Arg_ and IgG in PBS. We attribute the spot-like pattern observed to the size of the pores within the extracellular matrix of the brain, which are approximately 60 nm in diameter. This would limit the diffusion of large agents within the brain parenchyma once delivered across the BBB. Notably, in future work, employing rapid-short pulses of ultrasound could be used to increase the uniformity of delivery.^81,82^

## Conclusions

In this work, we present a thermodynamic-guided approach and outline the key design considerations for utilizing ILs as building blocks for constructing complex formulations of IgG-IL-AuNPs. By modulating hydrogen bonding and electrostatic interactions of biocompatible ILs and amino acids of diverse physicochemical and structural properties, the IgG molecules were conjugated to the IL-AuNP surfaces, yielding systems offering spontaneous self-assembly, suppressed aggregation, and enhanced structural, thermal, and thermodynamic stability. Improved *in vivo* FUS-mediated delivery and accumulation in the brain were demonstrated for the complex formulation containing IgG-[Cho]­[Cl]-AuNPs, trehalose, histidine, and arginine molecules. The developed design approach eliminates the requirements of structural modifications of the IgG molecules and enables streamlined functionalization of nanocarrier surfaces, which are challenging to modify via traditional covalent conjugation strategies. We believe that the engineered IgG-IL-AuNPs offer novel opportunities as powerful therapeutic delivery platforms, and our study will advance the nascent field of IL nanotechnology. The insight provided can inform the rational design of IL-nanocarriers to ultimately create ideal platforms for a broad range of delivery applications.

## Materials and Methods

### Materials

[Cho]­[DHP] was purchased from IoLiTec-Ionic Liquids Technologies GmbH (Heilbronn, Germany). [Cho]­[Cl], [Cho]­[OAc], trehalose, l-arginine, l-lysine, l-proline, l-phenylalanine, l-serine, l-cysteine, l-glutamic acid, l-histidine, 40 nm AuNP stabilized suspension in 0.1 mM PBS, and IgG-FITC from human serum (20 mg/mL) were purchased from Sigma-Aldrich Company Limited (Gillingham, Dorset, UK). All chemicals were stored as recommended and used without further purification.

### Methods

#### Preparation of Complex Formulations

Stock solutions of [Cho]­[Cl], [Cho]­[DHP], and [Cho]­[OAc] and each formulation buffer were prepared in ultrapure water (ELGA LabWater, High Wycombe, UK) in a glass vial (Thermo Fisher Scientific Inc., Waltham, MA, USA), as reported prior.^10,26^ Formulation buffers included 15 mM histidine HCl, 120 mM trehalose, and 75 mM of each of the l-arginine, l-lysine, l-glutamic acid, l-proline, l-serine, l-cysteine, and l-phenylalanine. For electrostatic conjugation, previously reported methodology was adapted and employed.^24,49,83–86^ The procedure was performed at 25 °C, utilizing Protein LoBind Eppendorf tubes (Eppendorf, Stevenage, UK), resistant to protein binding.^87^ AuNPs, as purchased, were washed and resuspended in the desired IL,^84,86^ and the solution was mixed at 500 rpm for 10 min, achieving an IL:AuNP ratio of 1:1%w/v.^49^ IgG-FITC was incubated, mixed at 100 rpm with the select formulation buffer for 10 min, added to the IL-AuNPs, and then stirred at 200 rpm for 15 min, followed by washing and resuspension in the select formulation buffer, for an IgG:formulation buffer:IL-AuNPs ratio of 1:17:51%w/v. The centrifugation-based method^88^ was avoided to limit shear force, which was found to increase aggregation propensity of the IgG-IL-AuNP systems (Table S5). For each system lacking an IL, the methodology described was also followed with the IL omitted. Finally, the pH of each complex formulation was adjusted to 6.5 by dropwise addition of hydrochloric acid and sodium hydroxide, confirmed via pH measurements employing the pH electrode Mettler Toledo InLab Micro (WOLFLABS, Pocklington, York, UK). Once prepared, the complex formulations were immediately stored at 4 °C until measured.

#### Dynamic Light Scattering and Zeta Potential Measurements

For each complex formulation, DLS and zeta potential measurements were performed as previously described.^18^ Briefly, DLS measurements were performed to obtain the *D*_h_ and PDI values using a Zetasizer Nano ZS (Malvern Panalytical Ltd., Malvern, UK) at a 90° scattering angle, and zeta potential measurements were conducted using a Litesizer 500 (Anton Paar GmbH, Ostfildern, Germany). For each experiment, the average of three measurements is reported.

#### TEM Experiments

TEM micrographs were obtained employing a JEOL JEM-2100F transmission electron microscope (JEOL, Tokyo, Japan), fitted with a Gatan Prius SC 1000 camera (2 × 4k) (Gatan, Pleasanton, CA, USA). An aliquot of 5 μL of each complex formulation was placed onto a 45 s glow-discharged 200 square mesh carbon-coated copper grid (Agar Scientific, Essex, UK). The grids were blotted with Whatman filter paper after 1 min deposition and imaged using a 2% w/v uranyl acetate solution (Sigma-Aldrich, St. Louis, MO, USA) as a staining agent. The samples were imaged using low electron dose rates to avoid electron beam damage.

#### CD Spectroscopy Experiments and Calculated Thermodynamic Parameters

Temperature variable CD spectroscopy experiments were performed,^26^ and the thermodynamic parameters were derived from the CD measurements as previously outlined.^10^ Briefly, samples were heated from 25 to 95 °C, and sequential CD spectra were recorded at 5 °C intervals. The MRE values at 218 nm from smoothed MRE data (OriginLab Corporation, Northampton, MA, USA) were converted into a plot of protein fraction denatured (*f*_D_) at a given temperature using a two-state model of denaturation, assuming equilibrium between the native (N) and denatured (D) protein states, with equilibrium constant (*k*_D_). The CD signal at 218 nm was plotted against the temperature, yielding a sigmoidal plot. For the native state, the region at low-temperature *t* was approximated to a linear fit

$$y_{N}=a_{N}+b_{N}T$$

where *y*_N_ is the predicted CD signal of the native protein at *t* and *a*_N_ and *b*_N_ are the temperature independent intercept and gradient, respectively. The equivalent linear relationship between *y*_D_, the predicted CD signal of denatured IgG, and *t* was derived by calculating *a*_D_ and *b*_D_ from a linear fit at high *t* values. *y*_N_ and *y*_D_ were then calculated for all *t*. From here, the fraction denatured was calculated as

$$f_{D}=\frac{\left(y-y_{N}\right)}{\left(y_{D}-y_{N}\right)}$$

where *y* was the measured CD signal. This was used to calculate the *k*_D_ and Gibbs free energy of denaturation (Δ*G*_D_) as

$$K_{D}=\frac{f_{D}}{1-f_{D}}$$

$$\Delta G_{D}=RT\ln K_{D}$$

for ideal gas constant *r*. Δ*G*_D_ was plotted linearly against temperature in the transition region (−5 kJ mol^–1^ < Δ*G*_D_ < 5 kJ mol^–1^), which describes the behavior of Δ*G*_D_ around the denaturation temperature (*T*_m_). The *T*_m_ was calculated as the *T* value for which Δ*G*_D_ = 0, as well as estimation of the change in enthalpy (Δ*H*_m_) and entropy (Δ*S*_m_) of denaturation from

$$\Delta G_{D}=0=\Delta H_{m}-T_{m}\Delta S_{m}$$

#### FUS-Mediated *In Vivo* Delivery

The experimental protocols outlined herein were approved by the institutional animal facility committee and the UK Home Office regulatory establishments. Twelve female wild-type C57bl/6 mice (10–13 weeks old, 20.3 ± 0.9 g; Envigo, Huntingdon, UK) were used in this study. Mice were treated with FUS and intravenously injected microbubbles (SonoVue, Bracco, Milan, Italy) to deliver the developed complex formulations to the left hippocampus of the brain (*n* = 3) while using the right hippocampus as the ultrasound lacking control, following a previously described protocol.^82^ One MHz ultrasound pulses were emitted at a peak-negative pressure of 0.530 MPa with a pulse length of 10 ms and a pulse repetition frequency of 0.5 Hz, for a duration of 250 s, based on previous work.^81,82^ For these ultrasound treatments, a single-element, focused ultrasound transducer (center frequency of 1 MHz, focal depth of 60.5 mm, active diameter of 90 mm, and central rectangular opening of 30 mm × 70 mm H-198, Sonic Concepts, Bothell, WA, USA) was driven by a function generator (33500B, Keysight, Santa Rosa, CA, USA) through a power amplifier (2100L, Electronics and Innovation, Rochester, NY, USA) and a matching network. This setup had previously been calibrated using a needle hydrophone (diameter of 0.2 mm, Precision Acoustics Ltd.) showing an ultrasound beam with a lateral diameter of 2 mm, an elevational diameter of 1 mm, and an axial length of 20 mm defined by the peak-rarefactional pressure full width at half-maximum. Following the ultrasound treatment, mice were transcardially perfused and the brain of each mouse was extracted, sectioned, and imaged with a fluorescence microscope (10×; Zeiss Axio Observer, Oberkochen, Germany).^82^ FITC was excited at 470/40 nm, with emissions filtered at 525/50 nm. To quantify differences in the amount of fluorescence detected from the FITC, the NOD was measured for five sections in each brain, as previously described.^82^ For the distinct complex formulations, a two-sided Student’s *t* test was performed to determine whether variations in the NODs were significantly different (*P* < 0.05) between the mice brains. Analysis was conducted using MATLAB R2019b (Mathworks, Natick, MA, USA).

## Supplementary Material



## Notes

Table of abbreviations; chemical structures of the amino acids and the IL ions used; *D*_h_, PDI, and zeta potential values for each system; MRE spectra for each system; *T*_m_, Δ*H*, and Δ*S* values derived from the experimental CD spectroscopy data; TEM micrographs for additional select systems; and *D*_h_ and PDI values of the complex formulations prepared utilizing an alternative, adapted centrifugation-based method^88^ (PDF).
