# Supplementary material for: Design Principles for Engineering Ionic Liquid-Gold Nanoparticles for Therapeutic Delivery to the Brain
Source: ACS Nano. 2025 Jul 3;19(27):24806–16. doi: 10.1021/acsnano.5c02375 (PMC12269357; doi:10.1021/acsnano.5c02375)
Supplement: Supplementary file 1 [file nn5c02375_si_001.pdf]

Supporting Information

Design Principles for Engineering Ionic Liquid-Gold Nanoparticles for Therapeutic Delivery to the Brain

Talia A. Shmool,[a*] Laura K. Martin,[b] Andreas Jirkas,[a] Sophie V. Morse,[c,d] Claudia Contini,[e] Yuval Elani,[a] Jason P. Hallett[a]

[a] Department of Chemical Engineering, Imperial College London, South Kensington Campus, London SW7 2AZ, UK
[b] Department of Engineering Science, University of Oxford, Parks Road, Oxford OX1 3PJ, UK
[c] Department of Bioengineering, Imperial College London, South Kensington Campus, London SW7 2AZ, UK
[d] UK Dementia Research Institute at Imperial College London, White City Campus, London W12 0BZ, UK
[e] Department of Life Sciences, Imperial College London, South Kensington Campus, London SW7 2AZ, UK
* Email: t.shmool20@imperial.ac.uk

Table S1. List of abbreviations.

Abbreviation	Meaning
IL	Ionic liquid
IgG	Immunoglobin G
AuNPs	Gold nanoparticles
[Cho][OAc]	Choline acetate
[Cho][DHP]	Choline dihydrogen phosphate
[Cho][Cl]	Choline chloride
BBB	Blood-brain barrier
FUS	Focused ultrasound
Arg	Arginine
Cys	Cysteine
Lys	Lysine
Phe	Phenylalanine
Pro	Proline
Ser	Serine
FITC	Fluorescein isocyanate
DLS	Dynamic Light Scattering
CD	Circular Dichroism
TEM	Transmission Electron Microscopy
D_h	Hydrodynamic diameter
PDI	Polydispersity index
PBS	Phosphate buffered saline
MRE	Mean residue ellipticity
T_m	Melting temperature
ΔH	Change in enthalpy
ΔS	Change in entropy
NOD	Normalised optical density

Table S2: Hydrodynamic diameter (D_h), polydispersity index (PDI) in bold, and zeta potential values in italics are given for the IgG-AuNPs and IgG-IL-AuNPs in each formulation developed. Included are the ILs choline chloride ([Cho][Cl]), choline dihydrogen phosphate ([Cho][DHP]) and choline acetate ([Cho][OAc]). For a given formulation buffer, each consists of trehalose, histidine and a given amino acid ($F_{Amino\ acid}$), including arginine (F_{Arg}), lysine (F_{Lys}), glutamic acid (F_{Glu}), proline (F_{Pro}), cysteine (F_{Cys}), serine (F_{Ser}), phenylalanine (F_{Phe}). Chemical structures of formulation components are included.

**Amino acid**	**IL lacking**	**[Cho][Cl]** 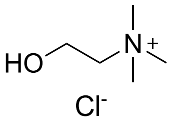	**[Cho][DHP]** 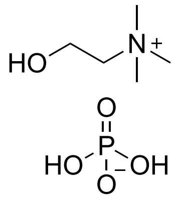	**[Cho][OAc]** 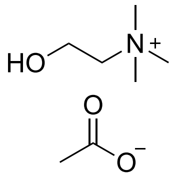
**Arginine (F_{Arg})** 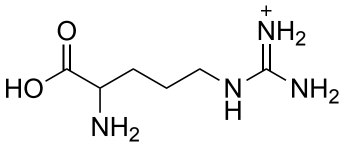	90 ± 20<br>**0.26 ± 0.02**<br>*-9 ± 2*	60 ± 1<br>**0.4 ± 0.1**<br>*-2.8 ± 0.4*	69 ± 2<br>**0.45 ± 0.02**<br>*-4 ± 1*	65 ± 5<br>**0.43 ± 0.08**<br>*-4 ± 2*
**Cysteine (F_{Cys})** 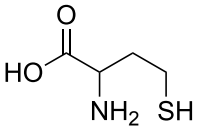	310 ± 50<br>**0.92 ± 0.05**<br>*-1.1 ± 0.3*	82 ± 6<br>**0.31 ± 0.04**<br>*-2.4 ± 0.4*	75 ± 2<br>**0.4 ± 0.1**<br>*-1.4 ± 0.1*	75 ± 2<br>**0.4 ± 0.1**<br>*-1.9 ± 0.1*
**Glutamic acid (F_{Glu})** 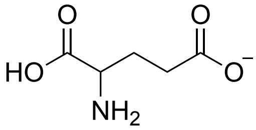	320 ± 90<br>**0.93 ± 0.07**<br>*7.1 ± 0.2*	78 ± 3<br>**0.30 ± 0.03**<br>*1.7 ± 0.3*	83 ± 5<br>**0.26 ± 0.04**<br>*2.4 ± 0.8*	72 ± 1<br>**0.30 ± 0.03**<br>*4.8 ± 0.1*
**Lysine (F_{Lys})** 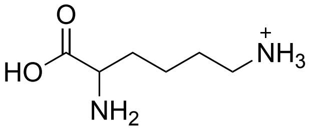	190 ± 50<br>**0.6 ± 0.2**<br>*-13.2 ± 0.2*	71 ± 3<br>**0.52 ± 0.06**<br>*-5 ± 1*	78.3 ± 0.5<br>**0.55 ± 0.08**<br>*-3.2 ± 0.7*	78 ± 3<br>**0.49 ± 0.01**<br>*-4.3 ± 0.6*
**Proline (F_{Pro})** 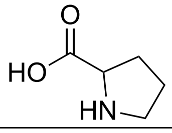	110 ± 20<br>**0.8 ± 0.2**<br>*-8.8 ± 1.3*	73 ± 2<br>**0.40 ± 0.04**<br>*-3.7 ± 0.2*	64 ± 4<br>**0.35 ± 0.07**<br>*-2.1 ± 0.1*	70 ± 1<br>**0.51 ± 0.04**<br>*-1.4 ± 0.3*
**Phenylalanine (F_{Phe})** 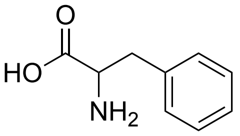	340 ± 40<br>**0.7 ± 0.3**<br>*-3.1 ± 0.8*	76 ± 1<br>**0.26 ± 0.03**<br>*-2.9 ± 0.1*	82 ± 4<br>**0.28 ± 0.04**<br>*-1.8 ± 0.2*	78 ± 3<br>**0.30 ± 0.07**<br>*-2.1 ± 0.9*
**Serine (F_{Ser})** 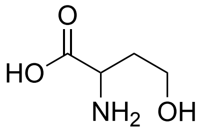	120 ± 10<br>**0.49 ± 0.05**<br>*-1.6 ± 0.8*	78 ± 2<br>**0.27 ± 0.04**<br>*1.1 ± 0.1*	75 ± 2<br>**0.28 ± 0.01**<br>*0.6 ± 0.1*	79.3 ± 0.4<br>**0.29 ± 0.07**<br>*1.3 ± 0.5*

Table S3. Hydrodynamic diameter (D_h), polydispersity index (PDI) and zeta potential values for gold nanoparticles (AuNPs) in phosphate buffered saline (PBS), immunoglobulin G (IgG)-fluorescein isothiocyanate (FITC) antibody in water and phosphate buffered saline (PBS), and IgG-AuNPs in PBS.

System	D_h	PDI	Zeta potential (mV)
AuNPs in PBS	43 ± 5	0.24 ± 0.02	-39 ± 3
IgG-FITC in water	75 ± 20	0.6 ± 0.3	2.3 ± 0.8
IgG-FITC in PBS	22 ± 2	0.4 ± 0.3	-1.9 ± 0.9
IgG-AuNPs in PBS	170 ± 20	0.62 ± 0.06	-20.1± 0.7

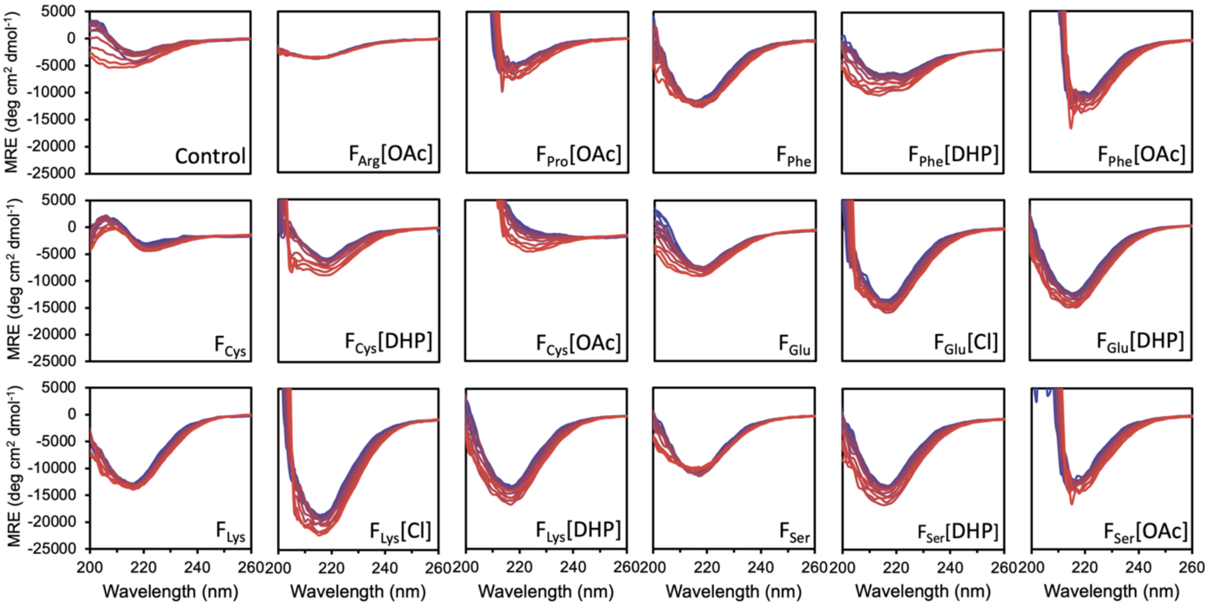

Figure S1. Mean residue ellipticity (MRE) spectra for the systems including IgG-AuNPs and IgG-IL-AuNPs. MRE spectra are derived from the temperature variable CD data measured from 200 to 260 nm, with temperature increasing from 25 °C (blue) to 95 °C (red) in 5 °C increments. Also shown is IgG in PBS (control). Each complex formulation shown consists of IgG, AuNPs, trehalose, histidine, and a select amino acid including arginine (Arg), cysteine (Cys), glutamic acid (Glu), lysine (Lys), phenylalanine (Phe), proline (Pro), and serine (Ser). The ILs [Cho][Cl], [Cho][DHP], and [Cho][OAc], and complex formulations lacking these are shown.

Table S4. Melting temperature (T_m), change in enthalpy (ΔH) and entropy (ΔS) derived from experimental CD spectroscopy measurement data for IgG-AuNPs and IgG-IL-AuNPs.

**System**	$\boldsymbol{T_m}$ **(°C)**	$\boldsymbol{\Delta H}$ **(kJ/mol)**	$\boldsymbol{\Delta S}$ **(J/K/ mol)**
F_{PBS}	85.4 ± 0.3	62 ± 28	700 ± 300
F_{Arg}	83.7 ± 0.2	68 ± 3	810 ± 20
F_{Arg}[Cho][Cl]	84.7 ± 0.3	57.8 ± 0.5	682 ± 4
F_{Arg}[Cho][DHP]	82.2 ± 0.5	50 ± 10	620 ± 80
F_{Cys}	53 ± 1	13 ± 1	240 ± 20
F_{Cys}[Cho][OAc]	83.9 ± 0.7	60 ± 10	700 ± 70
F_{Cys}[Cho][Cl]	81.6 ± 0.4	61 ± 8	700 ± 100
F_{Cys}[Cho][DHP]	81.5 ± 0.4	63 ± 8	800 ± 100
F_{Glu}[Cho][Cl]	72 ± 1	70 ± 30	900 ± 300
F_{Glu}[Cho][DHP]	73.0 ± 0.6	35 ± 4	480 ± 30
F_{Phe}[Cho][Cl]	82.8 ± 0.5	60 ± 10	700 ± 200
F_{Phe}[Cho][DHP]	75.6 ± 0.4	39 ± 4	520 ± 50
F_{Pro}	80.4 ± 0.4	51 ± 6	640 ± 80
F_{Pro}[Cho][Cl]	80.9 ± 0.3	65 ± 10	800 ± 70
F_{Pro}[Cho][DHP]	74.8 ± 0.5	38 ± 5	500 ± 40
F_{Ser}[Cho][Cl]	79.5 ± 0.9	62 ± 10	800 ± 100

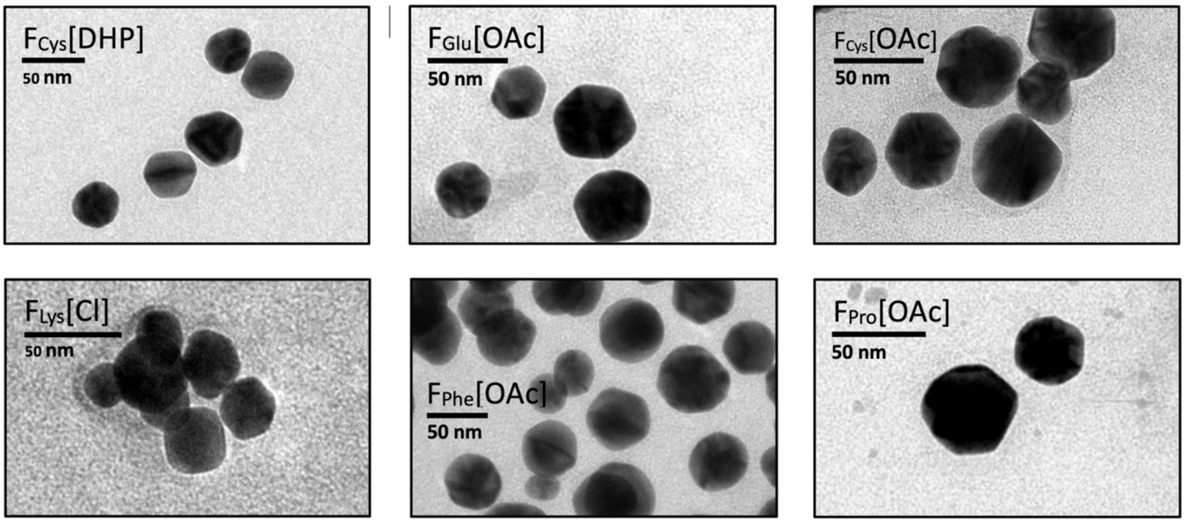

Figure S2. TEM micrographs of select complex formulations each including IgG-IL-AuNPs.

Table S5. D_h and PDI values for IgG-IL-AuNPs prepared utilising an adapted centrifugation-based method.[1] D_h and PDI values were found higher compared to our developed methodology, whereby centrifugation is omitted.

System	D_h	PDI
F_{Arg}[Cho][Cl]	92 ± 4	0.2 ± 0.0007
F_{Arg}[Cho][DHP]	79 ± 17	0.3 ± 0.06
F_{Arg}[Cho][OAc]	388 ± 49	0.9 ± 0.07
F_{Lys}[Cho][Cl]	259 ± 26	1 ± 0
F_{Lys}[Cho][DHP]	375 ± 92	0.9 ± 0.08
F_{Lys}[Cho][OAc]	467 ± 27	0.9 ± 0.02
F_{Glu}[Cho][Cl]	358 ± 89	0.9 ± 0.07
F_{Glu} [Cho][DHP]	888 ± 45	1 ± 0
F_{Glu} [Cho][OAc]	480 ± 22	1 ± 0.04
F_{Pro} [Cho][Cl]	154± 60	0.9 ± 0.2
F_{Pro} [Cho][DHP]	161 ± 4	0.6 ± 0.04
F_{Pro} [Cho][OAc]	103 ± 18	0.9 ± 0.1
F_{Cys} [Cho][Cl]	105 ± 19	0.9 ± 0.1
F_{Cys} [Cho][DHP]	908 ± 35	1 ± 0.04
F_{Cys} [Cho][OAc]	240 ± 111	0.8 ± 0.09
F_{Ser} [Cho][Cl]	146 ± 23	0.6 ± 0.06
F_{Ser} [Cho][DHP]	74 ± 44	0.9 ± 0.1
F_{Ser} [Cho][OAc]	182 ± 37	0.8 ± 0.2
F_{Phe} [Cho][Cl]	97 ± 4	0.3 ± 0.02
F_{Phe} [Cho][DHP]	309 ± 78	1 ± 0
F_{Phe} [Cho][OAc]	139 ± 48	0.8 ± 0.3

Reference

(1) Liang, Z.; Zhang, J.; Wang, L.; Song, S.; Fan, C.; Li, G. A Centrifugation-Based Method for Preparation of Gold Nanoparticles and Its Application in Biodetection. *Int. J. Mol. Sci.* **2007**, *8* (6), 526-532.
